# Restoration of an Upper Anterior Tooth in an Adolescent with Autism Spectrum Disorder—A Student Case Report

**DOI:** 10.3390/children7110237

**Published:** 2020-11-19

**Authors:** Max Diekamp, Leonie Jenter, Andreas G. Schulte, Oliver Fricke, Peter Schmidt

**Affiliations:** 1 Integrated Clinical Course, Dental School, Witten/Herdecke University, 58455 Witten, Germany; max.diekamp@uni-wh.de (M.D.); leonie.jenter@uni-wh.de (L.J.); 2 Department of Special Care Dentistry, Dental School, Witten/Herdecke University, 58455 Witten, Germany; andreas.schulte@uni-wh.de; 3 Department of Child and Adolescent Psychiatry, Psychotherapy and Child Neurology, Gemeinschaftskrankenhaus Herdecke, 58313 Herdecke, Germany; o.fricke@gemeinschaftskrankenhaus.de; 4 Department of Child and Adolescent Psychiatry, Witten/Herdecke University, 58455 Witten, Germany

**Keywords:** autism spectrum disorder, child, adolescent, dental treatment, SHCN, dental education, behavior facilitation

## Abstract

Background: Patients with autism spectrum disorder (ASD) or other mental or physical limitations experience an imbalance in the frequency of dental treatment as compared with the general patient population, in part, due to inadequate pre-graduate training of future dentists. Case presentation: This case report describes a successful anterior tooth restoration, in awake state, in a 15-year-old boy with early childhood autism. The procedure was carried out independently by students of dentistry within the scope of their integrated clinical training semesters. Desensitization sessions were used as a preparatory measure and elements of behavioral facilitation (tell-show-feel-feel-do) were applied during the treatment. Conclusions: To avoid discrimination of this group of patients in the provision and quality of dental care, a structured approach to the transfer of theoretical and practical knowledge in the field of special care dentistry is indispensable. To this end, treatment strategies for special care patients should be taught to pre-graduate dental students as a fundamental part of their university curriculum.

## 1. Introduction/Background

In the dental treatment of children and adolescents with early childhood autism, the dental team may be confronted with unusual challenges related to the peculiarities in social behavior that this group of patients may evince. In consequence, patients in this group, or in groups with other mental or physical limitations, experience an imbalance in the frequency of dental treatment as compared with the general patient population, in part, due to inadequate pre-graduate training of future dentists in special care dentistry. In the literature, there are only a few case reports that describe the dental treatment of patients with autism spectrum disorder (ASD) in all age groups (children, adolescents, and adults). It is, moreover, striking that most of these treatments were performed under general anesthesia [1,2,3,4,5,6,7,8,9,10,11,12].

Early childhood autism is equally commonly known as classic autism or Kanner autism (after Leo Kanner, a child and adolescent psychiatrist, who first described the condition in 1943) [13]. Children with this developmental disability often show particularities in social behavior, such as a lack of interest in social contact or avoidance of eye contact [14,15,16]. These children also frequently show stereotypical or repetitive behavioral patterns and impaired verbal communication abilities (e.g., lack of phonological articulation, echolalia), often in conjunction with some degree of intellectual disability [17,18,19]. In most cases, the first signs of early childhood autism can be recognized in the first months of life and can usually be diagnosed quite reliably from around the age of three [17,20,21].

Currently, the classification of autism is not yet globally uniform. While the WHO ICD-10 classification system includes three forms of autism (early childhood autism (F84.0), atypical autism (F84.1), and Asperger’s syndrome (F84.5), the American Psychiatric Association’s (APA) DSM-5 psychiatric classification system, which is dominant in the USA, in 2013, began grouping all forms of autism together in the category of autism spectrum disorders (ASD), in recognition of the fluid transitions between the different forms of autism [22]. According to the currently valid ICD-10 classification, the various forms of autism are considered to be subforms of pervasive developmental disorders (F84) [23]. In the ICD-11, which is expected to become valid as of 1 January 2022, the term ASD is also to be adopted (6A02) [24].

Each of the different forms of autism can be regarded to be a developmental disorder that cannot yet be cured, and each form has a different, and not yet fully understood, etiology. Although autism occurs in all age cohorts and in all ethnic and socioeconomic groups [17,25], its prevalence appears to vary by country and year of data collection. In a systematic review published in 2020 by Corridore et al., the authors reported prevalence rates between 1.4 and 146 per 10,000 children, with a slight upward trend over the last decades [26]. The data were, however, mostly based on studies from the USA, the UK, Brazil, and Arabia [14,27,28], with very little epidemiological data from Europe and Asia [28,29,30,31]. Across the board, these studies, with one exception [32], reported that males were at least three to four times more likely to be affected by ASD than females [26,33].

The present student case report, which also illustrates the goal that the Department of Special Care Dentistry is striving to achieve, aims to show that, if appropriate elements of behavior facilitation are taken into account, dental treatment of patients with ASD is possible without general anesthesia or sedation. Additionally, this case report would like to encourage dental students to acquire the skills necessary for the treatment of patients with ASD during their education in dentistry.

## 2. Case Presentation

### 2.1. Background/Treatment Plan

In May 2018, a 15-year-old boy with early childhood autism presented at the Department for Special Care Dentistry at Witten/Herdecke University. Although the teenager lived in an assisted living facility for children and young people with disabilities, his relationship with his family was good, especially with his mother. His mother also accompanied him, as his guardian at that time, during the actual treatment, by a dental student in clinical training, that we report on here. During the other appointments (regular check-ups, pretreatments, meetings), the patient was consistently accompanied by the same caregiver from the assisted living facility. According to both, the boy’s mother and his caregiver, he was not taking any medication regularly. In an assessment of the boy’s social interaction skills, it became clear that while his speech comprehension was good, verbal communication was not feasible because he did not speak. He could use a talker and construct two to three word sentences with it. In this way, he expressed wishes and interests in the context of leisure and family. Surprisingly, he showed little to no signs of refusal whatsoever concerning sounds, lights, and smells in the clinic. Perhaps also because an element of joy for him was water. Sounds or touching water had a calming effect.

**Figure 1:**
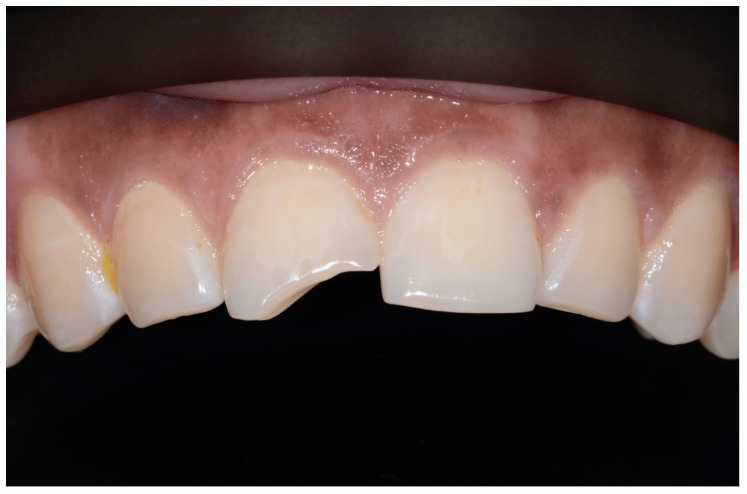
Initial situation, enamel-dentine fracture of tooth 11 of an autism spectrum disorder (ASD) patient. (©Max Diekamp).

**Figure 2:**
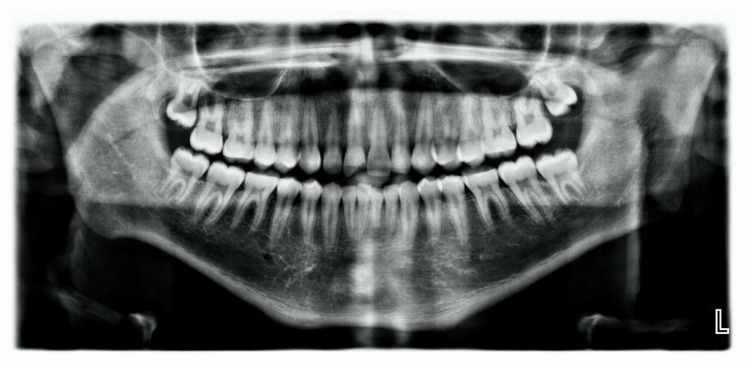
The ASD patient’s orthopantomogram shows a dentition free of caries and restorations, and the diagonally fractured incisal edge of tooth 11. No apical translucency or widened periodontal gap is visible. (©Witten/Herdecke University).

An intraoral examination showed that he had a complete set of caries-free permanent teeth. The only clinical finding was a coronal enamel-dentine fracture without pulp exposure in the area of the incisal edge of tooth 11 (Figure 1). There were no visible signs of a vestibular fistula. Further clinical examinations, which included percussion, palpation, and sensitivity testing were inconspicuous. Since the radiographic examination did not show translucency in the apical region of tooth 11 (Figure 2), an “uncomplicated crown fracture of tooth 11” was diagnosed. At the time of the initial examination in the Department for Special Care Dentistry (May 2018), there were no bruise marks on the patient’s face or other body parts and no signs of intraoral abnormalities such as bleeding, swelling, or injuries, for example, from a fall. There had been no way of establishing from the people in the boy’s social environment as to when and how the enamel-dentine edge of the tooth might have broken off. Obviously, the trauma to this tooth had occurred at some previous time and the cause could no longer be determined. The patient’s caregivers (residential caregiver and mother) were informed that restoration of tooth 11 was feasible. Although they welcomed the prospect of possible treatment, especially because of the poor aesthetics of the chipped tooth, they also pointed out, several times, that the general medical and social characteristics of the boy might make treatment difficult.

### 2.2. Therapy

A therapy plan was determined together with the patient’s mother and his caregiver, who were both also attachment figures for him. The proposal was to, first, adapt the patient to the dental setting before beginning with the actual restorative treatment. Initially, this was to be done in several short desensitization sessions in mostly familiar surroundings that were to be carried out by an experienced dentist, who was also a lecturer for the Department of Special Care Dentistry (P.S.). One of the patient’s attachment figures was to be present during these sessions (either the mother or the caregiver). If the desensitization sessions proved successful, and the patient tolerated intraoral examination and dental cleaning, the restorative measures were to be carried out in awake state by practicing elements of modified (or extended) behavioral facilitation (tell-show-feel-feel-do). Both the patient’s mother and his caregiver were informed about all aspects of the therapy and agreed to the treatment plan. They also consented to the patient being treated by supervised dental students in clinical training. According to plan, the supervising lecturer conducted several control and examination sessions, followed by a session with professional dental cleaning, with the aim of promoting patient cooperation. As part of the preparatory sessions, the patient was primed to also accept being treated by dental students. First, the student who was later to fulfill the role of “dental assistant” (L.J.) was, therefore, introduced to the patient during one of the preparation sessions. There, she performed a professional teeth cleaning for the patient. This measure also served to test if the patient was ready to accept being treated by other persons. At the next appointment, therefore, the patient was already familiar with this dental student, and then the student who was to be the dental operator (M.D.) for the tooth restoration was introduced to the patient as a “new” member. Furthermore, the patient was assured by P.S. and L.J., both of whom the patient was already familiar with, that he would also see them again during the restorative treatment session. After the supervising lecturer was of the opinion that the patient would be sufficiently cooperative for an invasive and time-consuming treatment in awake state, an appointment was made for the restoration of tooth 11 by a 5th year dental student in clinical training (M.D.). Because it is general practice in Germany for dentists to work together with dental assistants, dental students also take turns assisting each other while treating patients. In the present case, therefore, this role of dental assistant was undertaken by another 5th year dental student (L.J.). The therapy in the case reported here was performed during October 2019, with the student M.D. as the sole dental operator. During the restoration, the supervising dentist had only briefly been present in the treatment room to welcome the patient and to say goodbye to him, and, later, to check the finished restoration.

**Figure 3:**
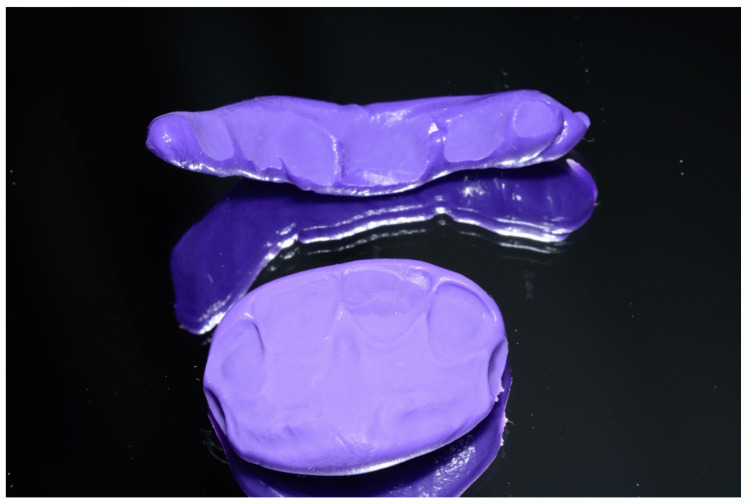
Layering key made of kneaded silicone (bottom), and kneading mass used to promote patient cooperation in the sense of the tell-show-feel-feel-do technique (top). (©Max Diekamp).

The aim of the operating dental student was to produce an aesthetically pleasing restoration of tooth 11 based on a standard approach for fillings of anterior teeth [34,35,36]. To this end, first, a palatal layer key made of kneadable silicone (Honigum-Putty, DMG Chemisch-Pharmazeutische Fabrik GmbH, Hamburg, Germany) was made. This required situation impressions of the anterior defect and the adjacent teeth, which were also made with kneadable silicone. Then, the silicone impressions were filled with hard plaster (dentostone^®^220, Dentona AG, Dortmund, Germany). Afterwards, a composite shade (Filtek XTE, 3M Espe GmbH, Seefeld, Germany) was used, chairside, to model an approximation of the final situation (so-called mock-up) on the hard-stone model. The young patient observed the procedure with great interest, throughout. In the spirit of promoting cooperation, the tell-show-feel-feel-do behavioral technique was used, and it was even possible to involve the patient in the manufacturing process. After the two dental students (operator and assistant) had shown the patient how to knead the silicone mass after its two individual components had been mixed together, he was shown how the palatal key was placed on the plaster model (tell, show, feel). The patient was also given a small portion of the silicone mass, which he attached (feel, do) to the front of the hard plaster model (vestibular side). Both parts of the silicone key (vestibular and palatal) could, thus, cure simultaneously. In keeping with the structured behavioral approach, the hardening process was observed together with the patient, and then he was allowed to visually and haptically inspect the cured silicone key (Figure 3).

**Figure 4:**
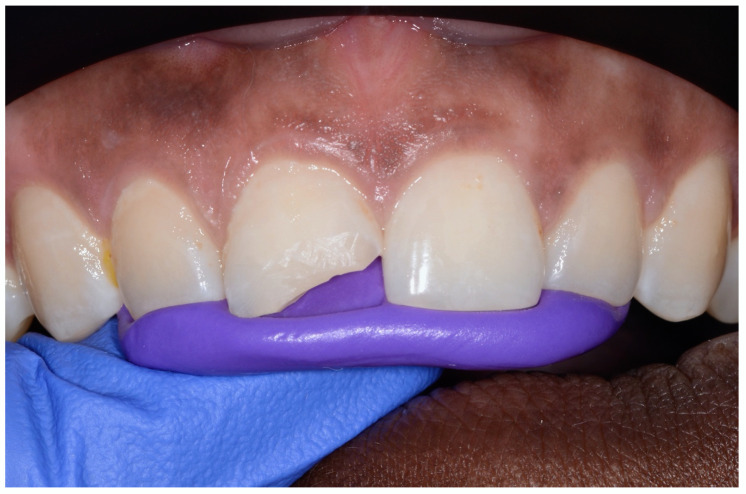
Fitting of the layering key after production of an enamel bevel in tooth 11. (©Max Diekamp).

The aim of the above-mentioned preparatory measures was to gain the patient’s cooperation in the subsequent intraoral treatment of his anterior tooth. After determining the tooth color (VITA A3), the enamel margins of the defect were first chamfered with rotating instruments (Diamant 863G and 833G, both green and red ringing, Hager & Meisinger GmbH, Neuss, Germany) (Figure 4). Palatinally, only the refraction of the enamel prisms was performed, while an approximately 2 mm wide bevel was prepared vestibularly. Because the defect was only medium sized and the rotating instruments were used only briefly, there was no necessity for local anesthesia. Furthermore, in agreement with the supervising lecturer, the operating dental student deliberately avoided using a rubber dam, as this procedure could easily have been too much for the patient to tolerate. Since treatment in anterior regions of the oral cavity is feasible with only relative drainage, cotton rolls and permanent suction were the drainage method of choice in this case. In a next step, the tooth surface was prepared for an adhesive bond with the composite resin restorative material using an enamel-etching technique. For this purpose, 36% phosphoric acid gel (DeTrey Conditioner 36, Dentsply Sirona GmbH, Bensheim, Germany) and a multi-bottle adhesive system (Optibond FL, Kerr GmbH, Biberach, Germany) were used.

**Figure 5:**
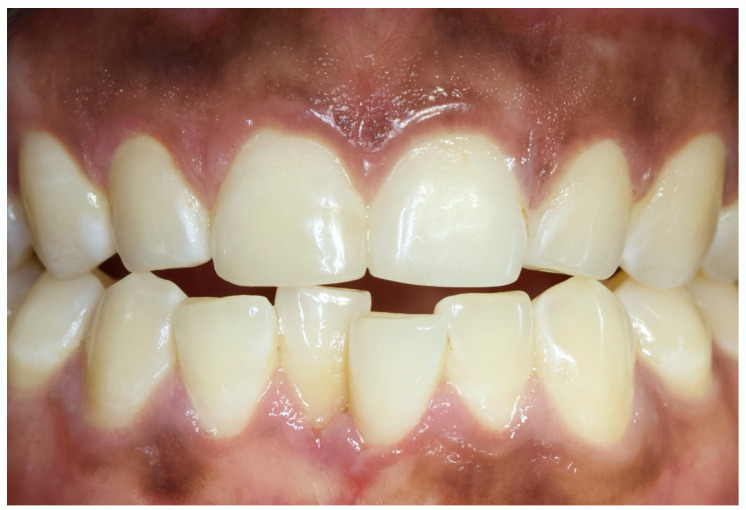
Control of the restoration of tooth 11 in the ASD patient after three months. (©Dr. Peter Schmidt).

The restoration filling was built up in partial steps using different composite materials (enamel mass, body mass, dentine mass-Filtex XTE). First, a thin layer of enamel mass was placed in the layering guide, adapted, and then light-cured, intraorally, to create a thin enamel shell on the palatal side. A small core consisting of body and dentin materials could, subsequently, be built up in this shell. Then, this core was also coated with enamel on the vestibular side. In a last step, the filling was finished with a carbide finisher (HM48LU 012, Hager & Meisinger GmbH, Neuss, Germany) and polished with polishing wheels (Sof-Lex™, 3M Espe GmbH, Seefeld, Germany) and ceramic polishers (9771f and 9771C, Hager & Meisinger GmbH, Neuss, Germany). The total time needed for the restoration per se was about 45 min. This time estimate did not include the time required for welcome and farewell of the patient and a short pre- and debriefing session, or time spent answering interim questions from the patient’s mother. Because the patient had become very restless during the final polishing process, high gloss polishing could not be completed during the treatment session and had to be done in a subsequent control session (Figure 5). The recall was set at a quarterly interval.

### 2.3. Consent for Publication

Written and signed consent to publish the images and the personal information about the patient in this case report was obtained from the mother of the patient (legal guardian till summer 2020) and the new state legal guardian (since summer 2020).

## 3. Discussion

This case report describes, for the first time, successful invasive dental treatment, in awake state, in an adolescent with early childhood autism, carried out independently by a team of dental students in the integrated clinical training phase of their curriculum. The team of authors is aware of only 12 other dental case reports in the international literature on patients with ASD [1,2,3,4,5,6,7,8,9,10,11,12]. Interestingly, the aspect of treatment carried out in our case has, however, not been described so far. Only one of the published case reports, which also describes tooth restoration in an adolescent with ASD after dental trauma, is comparable to the case we report here [10]. In contrast to our case, treatment in that case was, however, performed under general anesthesia.

The dental treatment of fully awake patients with ASD creates an exceptional situation for the dentist, due to the need to manage the specific social behavior that individuals in this patient population may display. In addition, it can be exceptionally difficult, or even impossible, for the dental operator and their team to interpret nonverbal cues (or lack of response) in regard to the patient’s well-being, perception of pain, and wishes or ideas (e.g., in regard to color, shape). Thus, the treatment conditions can be quite challenging for all involved [15,37]. The successful outcome of the treatment approach we report here was the result of meticulous attention to, and accommodation of, different aspects of the special behavior and communication peculiarities of the patient due to early childhood autism.

In our case, the extent of treatment was increased from session to session. Initially, several short preparatory sessions (controls, oral hygiene sessions) were held before the actual treatment appointment to promote the patient’s cooperation by giving him time to adapt to the dental setting and become comfortable with the surroundings. With ASD patients, it is important to create as much routine as possible and to confront the patient with as few unfamiliar things as possible [18,38,39]. Patients’ families can also support this approach from home by preparing the patient for the dental appointment, for example, by looking at pictures of the practice, the practice team, or the rudimentary procedure together with the patient. In our opinion, for every individual with ASD, a practitioner needs an individual plan dealing with peculiar sensitivities, social reinforcers, and socialization issues. Especially, in the dental setting, attention should be paid to keep as many aspects as possible constant, for example, same treatment team, same cuddly toy, same routines (e.g., always greetings first, then mouth protection, glasses, hand disinfection, gloves, etc.). It has proved helpful to record this kind of specific behavioral information in the patient file for reference. In our Department for Special Care Dentistry, this kind of documentation is established procedure and, as such, is taught to dental students as part of the curriculum. In the reported case, constancy was achieved by keeping the surroundings the same (the treatment rooms and waiting areas are very similar in most areas of the clinic) and by making sure that the patient was, as far as possible, always accompanied by the same persons. In addition, certain elements of behavior facilitation (e.g., positive reinforcement, tell-show-feel-feel-do) were practiced [17,19,40]. This constancy aspect was, however, slightly modified in regard to the treatment team. Although the use of behavioral elements usually favorably disposes patients to cooperate during treatment, they may still sometimes be overtaxed [25]. In addition, in our case, possible signs of excessive demands on the patient were observed shortly before completion of the restorative therapy. This overtaxation was likely less related to the nature of the practiced behavioral elements than to the increase in treatment time through their implementation. As described above, the invasive treatment itself only lasted about 45 min. During the final treatment step (high gloss polishing of the restoration), the patient became restless and his overall compliance diminished. According to a guideline from the textbook Kinderzahnmedizin (Paediatric Dentistry)*,* the duration of dental treatment in children should normally not exceed the length of three minutes per year of age [41]. Although the chronological age of the adolescent patient in our case was 15 years at the time of treatment, this rule of thumb for children was still applicable on grounds of his intellectual impairment. Therefore, the recommended maximum duration of treatment for our patient was 45 min (15 years × 3 min) per session. It is important to keep such simple parameters for the determination of the appropriate duration of treatment in mind, also in cases with adolescents or adults with disabilities. Keeping an eye on the length of a treatment session is a task that can well be taken over by one of the persons accompanying a patient, for example, by setting a timer or the alarm function on a smartphone. Furthermore, postponing certain treatment steps, such as high gloss polishing, to a second, but timely, session should be considered if there are cues that the patient is feeling overtaxed and may need adjustment of the original treatment plan. The ability to detect these signs early on does, however, require a certain amount of experience in dealing with patients from such groups.

It is useful to mention another decisive parameter in this dental medical context. Petrova et al. (2014) described the so-called “level of functioning” in children with impairments [42]. Their study highlighted six different areas of abilities in children, such as listening, understanding, or speaking. Even in patients without limitations, communication between dentist and patient before, during, and after treatment is key to the production of an aesthetically high-quality restoration of an anterior tooth. In people with early childhood autism, verbal communication (spoken language) skills are, however, often impaired or inadequately developed from early childhood on [17,32,40]. Therefore, the “level of functioning” in this area would be considered to be very low in such children. This was also the case in regard to our patient’s language level. Although he had a good understanding of speech, he was unable to speak or produce sounds or words. Due to this circumstance, individual elements of behavior facilitation had to be performed primarily on a visual-haptic level, for example, kneading the silicone mass (Figure 3). Similar approaches, with a focus on visual parameters as a means of communication, are also described in the literature [17,32,40].

In the assessment of the patient’s dental status, it was notable that his teeth were free of caries. Restoration of tooth 11, on which we report here, was the only treatment measure needed. Currently, there is controversial discussion in the literature whether persons with ASD have an increased prevalence of caries, periodontitis, and trauma as compared with persons of the same age in the general population [18,43]. An association between dental trauma and ASD could not be established [43]. While some studies have shown an increased prevalence of caries or gingivitis in children with ASD [44,45,46], other studies have shown that cohorts of patients with ASD have less (or no) caries experience and less periodontal disease than cohorts of patients without ASD [18,47,48,49]. Therefore, it is likely that not every type of impairment must lead to an increased prevalence of caries, periodontitis, or trauma [43]. The clinical experience of the co-authors shows that a low caries experience is also the result of good oral hygiene and a balanced diet. Among persons with ASD, there is a subgroup who, as a result of unbalanced dietary habits, already have multiple caries defects in adolescence age [50]. Both of these factors, however, strongly depend on the quality of care and support provided in patients’ homes. Unfortunately, as was observed in a number of studies from different countries, this type of care generally still needs to be improved, in light of the fact that the oral health status of persons with intellectual disability, or children attending special needs schools because of various disabilities, was, on average, shown to be poorer than that of persons of the same age in the general population [51,52].

In Germany, where dentistry is a five+ year course of integrative university study that encompasses both theoretical and practical training and culminates in a state administered licensing examination, only a few dental faculties specifically educate their dental students to also be able to provide dental care for patients with special needs on a professional level. In contrast, at Witten/Herdecke University, dental students are not only offered lectures on special care dentistry, but they are also obliged to assist in the dental therapy of persons with disabilities within the scope of integrated clinical courses. In addition, the students are also given the opportunity to treat patients with disabilities under the guidance and supervision of lecturers. This offer, which is regularly accepted by students, is made possible by the fact that the first and, so far, only chair for Special Care Dentistry in Germany began its activities at the Witten/Herdecke University in 2015. Thus, the dental students receive a structured education in the field of special care dentistry during four years of the five-year curriculum [53]. From the chair for Special Care Dentistry own unpublished data, it can be seen that a good third of the patients in the Department for Special Care Dentistry are patients diagnosed with ASD.

In light of the fact that the Federal Republic of Germany ratified the UN Convention on the Rights of Persons with Disabilities (UN-BRK) in 2009, the necessity of establishing several more such chairs should be undisputed [54]. The German licensing regulations for dentistry from 1955, which apply prospectively until 2020 (due to COVID-19 pandemic the ratification is delayed), contain no reference to dentistry for persons with disabilities [55]. With this fact in mind, the results of a questionnaire-based survey conducted among dentists from two different German regions in regard to special care dentistry are, therefore, not surprising: About 85% of the participants reported that they felt that they had been poorly prepared for the dental treatment of people with disabilities during their studies [56,57]. In the authors’ view, education of undergraduate students in special care dentistry is important for two reasons. The first reason is that various studies have shown that specific pre-graduate training in special care dentistry, or more particularly the lack thereof, later significantly affects the willingness of licensed dentists to treat children and adults with disabilities [15,38,39,42,58,59,60]. Secondly, it is stated in Section 3 (3) of the Constitution of the Federal Republic of Germany, which expresses equality before the law, “No person shall be disfavoured because of disability.” In other words, people with disabilities have, and must have, the same right to the same level of medical care as people without disabilities.

## 4. Conclusions

The different forms, for example, early childhood autism, and graduations of autism across the spectrum of this disability may necessitate adaptive strategies to allow dental treatment of this patient population in an awake state.

Moreover, in addition to practical skills and knowledge of treatment procedures in special care dentistry, a sound theoretical knowledge base is also essential for the dental team, for example, in regard to the specifics of certain types of disabilities. So far, this kind of knowledge is not standardly being taught to dental students as part of the dental school curriculum at all German universities. In the spirit of the UN Convention on the Rights of Persons with Disabilities, universities should strive to develop teaching concepts in the field of special care dentistry [54,61]. Such steps could encourage, and enable, dental students or young licensed dentists to take on the special challenges of caring for this group of patients.

### Practical Application

The authors believe the following basic principles to be crucial for successful dental treatment of these patients:

1. Creation of a dental environment that meets the psychological and physical needs of people with (early childhood) autism.
2. Inclusion of visual-haptic elements as part of the behavioral facilitation before and during dental treatment.
3. Choice of a sufficiently long treatment period so that the patient can get used to the dental setting in several short preparatory sessions prior to invasive treatment.
4. Adaptation of the duration of each treatment session to a length suitable for the patient, based on the patient’s age and the expected stress level of the upcoming preparation or treatment step.
